# Reply to Chen et al., “Methodological considerations for the establishment of epidemiological cutoff values for *Sporothrix* species using CLSI broth microdilution”

**DOI:** 10.1128/aac.00566-26

**Published:** 2026-06-24

**Authors:** Amanda R dos Santos, Alexandro Bonifaz, Amanda Bombassaro, Ana Caroline de Sá Machado, Anderson M. Rodrigues, Andrew M. Borman, Arunaloke Chakrabarti, Anuradha Chowdary, Bruno Rediguieri, Elizabeth M. Johnson, Ferry Hagen, Flavio Queiroz-Telles, Gloria M. Gonzalez, Guillermo Garcia-Effron, Isabella Dib Ferreira Gremião, Jacques F. Meis, Juliana Possato Fernandes Takahashi, Luana Pereira Borba dos Santos, Marcia S. C. Melhem, Paola Cappellano, Raimunda Sâmia Nogueira Brilhante, Sandro Antonio Pereira, Sarah Santos Gonçalvez, Sarah Kidd, Sean X. Zhang, Shivaprakash Rudramurthy, Sonia Rozental, Susana Cordoba, Theun de Groot, Wei Liu, Nathan P. Wiederhold, Eelco F. J. Meijer, Shawn R. Lockhart, Philippe J. Dufresne

**Affiliations:** 1 Departamento de Análises Clínicas e Toxicológicas, Faculdade de Ciências Farmacêuticas, Universidade de São Paulo 67781, São Paulo, Brazil; 2 Mycotic Diseases Branch, Centers for Disease Control and Prevention 1242, Atlanta, Georgia, USA; 3 Dermatology Service, Mycology Department, Hospital General de México, "Dr. Eduardo Liceaga" 61575, Mexico City, Mexico; 4 Department of Public Health, Federal University of Paraná 28122, Curitiba, Brazil; 5 Radboudumc-CWZ Center of Expertise for Mycology, Canisius-Wilhelmina Hospital (CWZ) 6030 , Nijmegen, the Netherlands; 6 Fundação Oswaldo Cruz (Fiocruz), Laboratório de Pesquisa Clínica em Dermatozoonoses em Animais Domésticos (Lapclin-Dermzoo), Instituto Nacional de Infectologia Evandro Chagas (INI) 663758 , Rio de Janeiro, Rio de Janeiro, Brazil; 7 Department of Microbiology, Immunology, and Parasitology, Discipline of Cellular Biology, Federal University of Sao Paulo (UNIFESP) 28105, São Paulo, Brazil; 8 National Institute of Science and Technology in Human Pathogenic Fungi, São Paulo, Brazil; 9 UK Health Security Agency National Mycology Reference Laboratory, Bristol and MRC Centre for Medical Mycology, University of Exeter 3286, Exeter, United Kingdom; 10 Doodhadhari Burfani Hospital and Research Institute, Haridwar, India; 11 Medical Mycology Unit, Department of Microbiology, Vallabhbhai Patel Chest Institute, University of Delhi 28742, Delhi, India; 12 Department of Pathology, Universidade Federal do Espírito Santo (UFES) 28126, Vitória, Espírito Santo, Brazil; 13 Department of Medical Microbiology, University Medical Center Utrecht, Utrecht, the Netherlands; 14 Faculty of Medicine, Autonomous University of Nuevo León 103563, Monterrey, Mexico; 15 Consejo Nacional de Investigaciones Científicas y Tecnológicas (CONICET), Universidad Nacional del Litoral 28247, Santa Fe de la Vera Cruz, Santa Fe, Argentina; 16 Excellence Center for Medical Mycology (ECMM), Institute of Translational Research, University of Cologne 14309, Cologne, Germany; 17 Centro de Patologia, Núcleo de Patologia Quantitativa, Instituto Adolfo Lutz 89119, São Paulo, Brazil; 18 Laboratório de Biologia Celular de Fungos, Centro de Pesquisas em Medicina de Precisão, Universidade Federal do Rio de Janeiro (UFRJ) 28125, Rio de Janeiro, Rio de Janeiro, Brazil; 19 Faculdade de Medicina, Universidade Federal do Mato Grosso do Sul, Campo Grande, Mato Grosso do Sul, Brazil; 20 Laboratório de ainvestigação Médica, Hospital das Clinicas, Faculdade de Medicina, Universidade de São Paulo 28133, São Paulo, Brazil; 21 Microbiology Section, Grupo Fleury 89577, São Paulo, São Paulo, Brazil; 22 Department of Pathology and Legal Medicine, School of Medicine, One Health Microbiology Laboratory, Postgraduate Program in Medical Microbiology, Postgraduate Program in Medical Sciences, Federal University of Ceará 28121, Fortaleza, Ceará, Brazil; 23 National Mycology Reference Centre, SA Pathology 10107, Adelaide, Australia; 24 Department of Pathology, Johns Hopkins University School of Medicine & Johns Hopkins Hospital 1500, Baltimore, Maryland, USA; 25 Department of Medical Microbiology, Post Graduate Institute of Medical Education and Research 685007, Chandigarh, Chandigarh, India; 26 Mycology Department of INEIA ANLIS “Dr. C. G. Malbran”, Buenos Aires, Argentina; 27 Peking University First Hospital 26447, Beijing, Beijing, China; 28 Department of Pathology and Laboratory Medicine, The University of Texas Health Science Center at San Antonio 14742, San Antonio, Texas, USA; 29 Laboratoire de santé publique du Québec, Institut National de Santé Publique du Québec (INSPQ) 54470, Sainte-Anne-de-Bellevue, Quebec, Canada; University Children's Hospital Münster, Münster, Germany

## REPLY

We thank Chen et al. for their correspondence (1) and interest in our recent publication, “Antifungal susceptibility profile of clinically relevant species of the genus *Sporothrix:* establishment of Epidemiological Cutoff Values (ECVs) according to CLSI broth microdilution methodology,” published in *Antimicrobial Agents and Chemotherapy*.

Overall, it appears that we are in agreement on most elements that are put forward and appreciate the opportunity to clarify several points raised in their letter.

Regarding the comment on the profound implication of testing *Sporothrix* at 35 °C, which could results in asynchronous yeast phase transition—most notably for itraconazole ECV at 4 µg/mL for *S. brasiliensis* and may represent methodological artifacts that overestimate true wild-type upper limits, we welcome the discussion and, as indicated in our manuscript, we agree that testing *Sporothrix* spp. in the mold phase using the current CLSI M38 broth microdilution methodology (2), in which the initial primary culture and conidia are incubated at 35°C, can induce asynchronous yeast-phase transition and may contribute to increased interlaboratory variability for certain antifungal agents.

However, it must be emphasized that this still remains a working hypothesis. The incomplete yeast transition is just one of the possible hypotheses to explain interlaboratory variation, which also includes variation in reading/interpretation; differences in panel preparation; paradoxical growth of some azoles or even antifungal precipitation of antifungals with poor aqueous solubility at higher concentrations (e.g., itraconazole and posaconazole). At present, limited data are available, and the extent and precise quantification of variation in minimum-inhibitory concentrations (MICs) between yeast and mold forms remain to be better defined.

Other studies and referenced work in our manuscript (3–5) suggest that lower MICs are obtained with yeast form for most antifungals, such as itraconazole, which undermines the hypothesis put forward by Chen et al. and would hypothetically lead to a lower ECV than the one that was determined.

This is also corroborated by some of the data submitted as part of our study, where a few laboratories also submitted, in parallel, MICs generated with the pure yeast form. We determined that testing of the yeast form resulted in the same or a 1 to 2 dilution reduction of the modal MIC (Table 1) for the majority of antifungals. However, this was not the case for voriconazole, which had significantly lower modal MICs (up to four or five dilutions lower). Another exception was observed for *S. schenckii* when tested in the yeast form against terbinafine, where we found a modal MIC of four dilutions higher (+4), when compared to the modal MIC for the *S. schenckii* mold form, to terbinafine. These seem to indicate that the MIC response for yeast and mold forms also varies according to antifungal and *Sporothrix* species (6) or strains (7, 8).

**TABLE 1:** Difference in yeast modal MICs versus mold modal MICs for laboratories that tested both morphologies in our study**^*a*^**

	*S. brasiliensis*	*S. schenckii*	*S. globosa*
Amphotericin	= (one lab)	= or −1 (two labs)	−2 (one lab)
Itraconazole	−2 (one lab)	= (two labs)	−2 (one lab)
Posaconazole	−2 (one lab)	= or −1 (two labs)	−1 (one lab)
Voriconazole	−5 (one lab)	−2 or −4 (two labs)	−4 (one lab)
Terbinafine	No yeast data	+4 (one lab)	No yeast data

^*a*^ – (number of labs in parentheses).

We agree that a more systematic evaluation of the impact of testing as yeast or mold forms is needed and that testing at 30°C may prevent the mold-to-yeast conversion while also being convenient for most laboratories. In that event, ECVs will need to be reassessed according to the new methodology and the M38 method modified for *Sporothrix*.

Regarding the comment of the concern for the tentative terbinafine ECV of 2 μg/mL proposed for *S. globosa* based on only 38 isolates, we appreciate the feedback on this tentative ECV and understand the concerns of Chen et al. Few laboratories have submitted MIC data for *Sporothrix* against terbinafine, and the ECV/tentative ECVs values were found to be quite widespread, with that of *S. brasiliensis* being at 0.12 µg/mL, while tentative ones from *S. globosa* and *S. schenckii* are at 1 and 0.5 µg/mL, respectively.

Presenting tentative ECVs (when MIC results are available for >35 isolates from ≥3 labs) is a novel approach we have introduced in this manuscript to give a preliminary baseline with available data when insufficient MICs are available to set a formal ECV according to CLSI M57 requirements (at least 100 isolates from at least three labs). We hope our readers understand that tentative ECVs are meant to be preliminary values to highlight initial tendencies observed but not to be used for formal surveillance or clinical interpretation of susceptibility testing at this point. This terbinafine and *S. globosa* tentative ECV, as for other tentative ECVs, is not to be published in the upcoming M57S CLSI supplement and could certainly change in the future as new data are collected.

The small number of isolates and laboratories sharing MIC data made it difficult to clearly assess if a bimodal distribution is present, as suggested by Chen et al. for *S. globosa* and terbinafine. Only three labs specifically submitted MIC data for this antifungal against *S. globosa,* with the bulk of MICs from a single laboratory (33 isolates/87% of the total). For the other two, one is in agreement with a single MIC at 0.5 µg/mL, while the other one shows four isolates at an MIC of 0.06 µg/mL (Table 2).

**TABLE 2:** MICs (µg/mL) obtained by three laboratories according to the CLSI M38 methodology for *S. globosa* and terbinafine in our study

	0.03	0.06	0.12	0.25	0.5	1	2	4	8	16
Lab 1				2	14	17				
Lab 2					1					
Lab 3		4								

More MIC data from a diverse group of laboratories are needed to define a formal ECV and confirm if *S. globosa* differs markedly from that for *S. schenckii* and *S. brasiliensis*. Still, our group believes the publication of this tentative ECV would be of interest to our community. It is important that laboratories do not overinterpret these tentative ECVs, or even the formal ECVs, as those are not backed with clinical outcome data and should not be used as a measure of clinical efficacy. We encourage laboratories to submit *S. globosa* terbinafine susceptibility data to the CLSI ECV working group to sort out this intriguing discrepancy and for the calculation of formal ECV.

The concern about the high interlaboratory data rejection rate (17%–30%) raised by Chen et al might be somewhat exaggerated. Twelve out of 18 antifungal combinations investigated in our study had ≥85% acceptance. A 20%–30% rejection rate is not that uncommon for some antifungals with molds for which MICs are more variable. Given the small number of laboratories that provided MIC data (3 to 11 at most here), exclusion of data from one or two laboratories can result in a high percentage of rejection. Nonetheless, we should focus our effort on those less concordant combinations for method verification and standardization.

There is currently no recommended *Sporothrix* QC or reference isolates in the CLSI M38M51S document. We agree with the authors that there is a dire need for *Sporothrix* reference strains, with defined MICs and proficiency programs for mold susceptibility testing. Selection of QC and reference strains according to the CLSI M23 protocol can be resource- and cost-prohibitive if not supported by antifungal drug sponsors. An alternate approach is likely needed for laboratories to compare and verify their MIC results for less common mold species. Potential reference strains for *S. brasiliensis* and *S. schenckii* are currently being tested by our group, and we hope to make those available to the community in the near future. Similar initiatives for *S. globosa* reference isolates would need to be launched.

We must emphasize that the primary objective of our study was to validate previously proposed epidemiological cutoff values (ECVs) for *Sporothrix* spp. for formal endorsement by the CLSI antifungal subcommittee. As such, CLSI M57 guidelines’ stringent criteria and statistical approach, designed to minimize the impact of interlaboratory methodological variability and reduce potential bias in ECV determination, were followed. ECV data presented here and associated MIC data were submitted to the antifungal subcommittee for careful review, approval, and vote. We believe that variability related to dimorphism did not significantly influence the MIC distributions used to establish these ECVs according to the current CLSI methodology and that our conclusions remain robust and supported by the proposed ECVs for amphotericin B, terbinafine, itraconazole, and posaconazole.

Importantly, the ECVs proposed in our study are not intended to guide clinical decision-making. The relationship between MIC values and clinical outcomes in sporotrichosis remains unclear, and favorable therapeutic responses have been reported even in cases involving isolates with elevated MICs (9). Rather, we propose that ECVs should be used as a tool for antifungal resistance surveillance. This is urgently needed in the context of the ongoing zoonotic epidemic in Brazil, where both cats and humans are treated with the same antifungal agents (10–12), raising concerns about the potential emergence and spread of antifungal resistance (13, 14),

We thank Chen et al. for their thoughtful comments and the Editor for the opportunity to respond.
